# Molecular Mechanisms of Heterosis and Its Applications in Tree Breeding: Progress and Perspectives

**DOI:** 10.3390/ijms252212344

**Published:** 2024-11-17

**Authors:** Zeyu Li, Yan Zhao, Keming Luo

**Affiliations:** 1 Key Laboratory of Eco-Environments of Three Gorges Reservoir Region, Ministry of Education, School of Life Sciences, Southwest University, Chongqing 400715, China; lzy20226040@whu.edu.cn (Z.L.); xndx17383027593@email.swu.edu.cn (Y.Z.); 2 Chongqing Key Laboratory of Forest Resource Innovation and Utilization, Integrative Science Center of Germplasm Creation in Western China (Chongqing) Science City, School of Life Sciences, Southwest University, Chongqing 400715, China

**Keywords:** heterosis, hybrid vigor, tree species, molecular mechanisms, epigenetics, genomics, marker-assisted selection

## Abstract

Heterosis, or hybrid vigor, refers to the phenomenon where hybrid progenies outperform their parents in traits such as yield and resistance. This phenomenon has been widely applied in plant breeding. Recent advances in high-throughput genomics have significantly advanced our understanding of heterosis. This review systematically summarizes the genetic, molecular, and epigenetic mechanisms underlying heterosis. Furthermore, we discuss recent advances in predictive methods for heterosis and their applications in improving growth rate, resistance to abiotic stresses, and wood yield in tree species. We also explore the role of tree genomics in unraveling the mechanisms underlying heterosis, emphasizing the potential of integrating high-resolution genomics, single-cell sequencing, and spatial transcriptomics to achieve a comprehensive understanding of heterosis from the molecular to spatial levels. Building on this, CRISPR-based gene-editing technologies can be employed to precisely edit heterotic loci, enabling the study of allele function. Additionally, molecular marker-assisted selection (MAS) can be utilized to identify heterotic loci in parental lines, facilitating the selection of optimal hybrid combinations and significantly reducing the labor and time costs of hybrid breeding. Finally, we review the utilization of heterosis in tree breeding and provide a forward-looking perspective on future research directions, highlighting the potential of integrating multi-omics approaches and emerging gene-editing tools to revolutionize tree hybrid breeding.

## 1. Introduction

Heterosis, also called hybrid vigor, refers to the phenomenon where hybrid progenies, produced by crossing parents with distinct genetic backgrounds, exhibit superior traits such as enhanced growth, yield, quality, and resistance to abiotic stresses compared to their parents. The study of heterosis has a long history, with research dating back several centuries. In 1763, hybrid tobacco plants were first observed to grow faster than their parent plants, providing early evidence of heterosis [1]. Later, in 1865, Gregor Mendel established the foundational laws of inheritance, laying the groundwork for understanding hybrid vigor [2]. Charles Darwin also contributed to the field, reporting that hybrid maize plants had greater average height compared to inbred progeny [3]. In 1908, George Shull formally introduced the term “heterosis” and applied it to maize breeding, leading to the development of the first hybrid maize varieties [4]. The application of heterosis in agriculture has since revolutionized the field. For example, maize yields increased by 8-fold [5], and wheat [6] and *Oryza sativa* [7] saw 10–20% increases in productivity. In recent years, the advent of high-throughput genomic technologies has enabled significant advancements in understanding the genetic and molecular mechanisms underlying heterosis. These technologies have revealed the complex regulatory networks driving hybrid vigor, providing powerful tools for dissecting its molecular basis.

The application of heterosis in tree species holds great promise for improving traits such as growth rate, disease and abiotic stress resistance, fruit quality, and wood yield. Eucalyptus trees doubled in volume, and poplar exhibited a 15% increase in growth rate [8]. However, tree species breeding presents unique challenges, including long generation times and the complexity of tree genomes. Despite these obstacles, recent progress in tree genomics and molecular biology has opened up new avenues for the study and utilization of heterosis in tree breeding programs.

In this review, we provide a comprehensive overview of the current understanding of the molecular mechanisms underlying heterosis, with a particular focus on the genetic basis, molecular regulation, and epigenetic modulation of heterosis. In addition, we summarize recent advances in predictive models for heterosis and highlight their application in tree species research. Finally, we discuss the potential of integrating cutting-edge technologies, such as CRISPR/Cas-9 genome editing and multi-omics approaches, to further elucidate the mechanisms of heterosis and accelerate the development of superior hybrid trees.

## 2. Mechanisms Underlying Heterosis

### 2.1. Genetic Hypotheses of Heterosis

**Figure 1:**
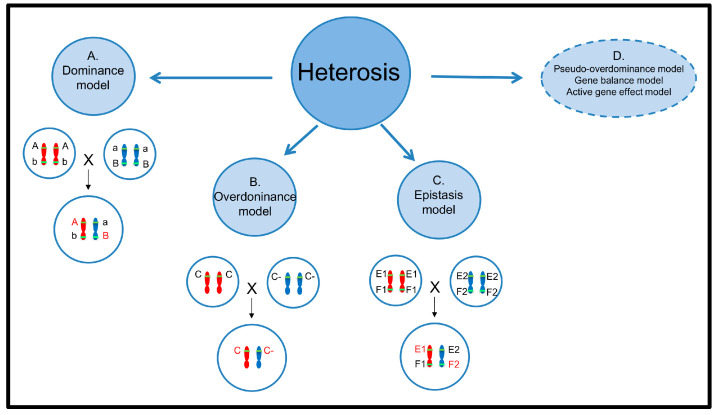
Genetic models of heterosis. (**A**). The dominance hypothesis. Dominant alleles (A/B) are more beneficial to F1 than recessive alleles (a/b) and accumulated dominant alleles, leading to heterosis. (**B**). The overdominance hypothesis. The interaction between heterozygous alleles (C/C-) at a locus is stronger than that of homozygous alleles (C/C or C-/C-), resulting in heterosis. (**C**). The epistasis hypothesis. The interaction between non-alleles (E1/F2) results in heterosis. (**D**). Other hypotheses to explain the genetic basis of heterosis: pseudo-overdominance hypothesis, gene balance hypothesis, active gene effect hypothesis.

Intensive studies on heterosis have been conducted for over a century, and various hypotheses have been proposed to explain its complex genetic mechanisms. The dominance hypothesis [9,10,11] suggests that dominant alleles are more beneficial to individual growth and development than recessive alleles (Figure 1A). Hybridization allows the F1 hybrids to accumulate dominant alleles from both parents, leading to heterosis [9,10,11]. In contrast, the overdominance hypothesis [3,12] proposes that the interaction between heterozygous alleles at a locus is stronger than that of homozygous alleles, resulting in heterosis in the offspring (Figure 1B). For example, heterozygous *pl* alleles in maize cause anthocyanin accumulation, and heterozygous *sft* alleles in tomato increase yield, demonstrating the overdominant effect [13,14]. The epistasis hypothesis [15] argues that interactions between non-allelic genes contribute to the formation of heterosis (Figure 1C). Evidence has shown that epistasis plays a crucial role in heterotic species such as *O. sativa* [16], maize [17], and tomato [18].

In addition to these classic quantitative genetic hypotheses, several other models (Figure 1D), such as the pseudo-overdominance hypothesis [19], the gene balance hypothesis [20], and the gene activation hypothesis [21], have been proposed to explain the genetic mechanisms of heterosis.

### 2.2. Molecular Mechanisms of Heterosis

Hybridization integrates distinct genomes combinations, often resulting in global changes in gene expression, including differential expression, allelic imbalance, and additive and non-additive expression patterns. Over the past two decades, advances in molecular biology, particularly high-throughput sequencing technologies, have accelerated our understanding of the molecular mechanisms underlying heterosis [22,23,24,25,26]. Advancements in genomic sequencing now enable researchers to dissect the genetic architecture of F2 populations derived from superior F1 hybrids, including genotypes, allele frequencies, and other genetic characteristics [22,27,28,29]. The “immortalized F2” population requires genotyping only once, but can be phenotyped multiple times. By comparing genetic information from the parental lines and exploring the emergence of new genetic combinations and their corresponding phenotypes, researchers can quantify the contributions of dominance, overdominance, and epistasis to heterosis [27,28,29]. For example, analysis of heterosis in 240 “immortalized F2” populations from the *O. sativa* hybrid Shanyou63 identified heterotic effects at 33 loci associated with four traits [27]. Further resolution of digenic interactions revealed that single-locus heterotic effects and dominance interactions largely explained the genetic basis of heterosis [27]. In “immortalized F2” populations of *Zea mays*, the various genetic effects were assessed using a high-density linkage map [29]. The results showed that, while dominance had a greater contribution to yield-related heterosis, both overdominance and epistasis also played significant roles in the formation of heterosis [29].

These findings suggest that multiple genetic mechanisms may concurrently drive the formation of heterosis.

Genome-wide association studies (GWAS) have identified numerous key genes related to heterosis for agronomic traits [22,30,31]. In *Arabidopsis thaliana*, for example, GWAS analysis of 200 F1 hybrids detected important candidate genes related to biomass, such as *WUSCHEL* and *ARGOS* [30]. Similarly, GWAS on elite hybrid *O. sativa* cultivars identified alleles associated with yield and other agronomic traits [22]. Genomic and phenotypic analyses of 1604 inbred maize lines revealed numerous candidate genes associated with heterosis, providing insights into improving agronomic traits [31]. Moreover, haplotype genome sequencing has shown that sequence divergence is a key factor contributing to heterosis formation [32].

Advances in transcriptomics have enabled the exploration of the molecular mechanisms of heterosis at the gene expression level. Non-additive expression (NAE) refers to gene expression in hybrids that deviates from the mid-parent value, and NAE has been shown to play a key role in heterosis for yield and organ size [33,34,35]. For instance, the glutathione S-transferase gene *OsGSTU26* has been identified as a contributor to yield and salt tolerance heterosis in *O. sativa* through NAE analysis [36]. NAE genes have been found to have divergent regulatory functions and, therefore, to play an important role in regulating domestication-related traits such as the gigantism of organ size [35]. In two hybrid poplars, plenty of NAE genes were associated with heterosis in vegetative growth, such as ground diameter and plant growth [37]. Allele-specific expression (ASE) describes the imbalanced expression of alleles inherited from the parents in hybrids, which is another mechanism explaining heterosis at the gene expression level [38]. In *O. sativa* hybrids, approximately 11–42% of alleles exhibit ASE, and these alleles are associated with partial or complete dominance [39,40]. In *Z. mays* hybrids, ASE was detected in up to 50% of alleles in seedlings, highlighting its significant role in contributing to heterosis [41]. Notably, the ASE patterns of many alleles vary across different developmental stages, tissues, or treatments, sometimes even reversing, contributing to dominance effects [32,42,43]. In hybrid *P. alba* × *P. tremula* var. *glandulose*, 30.1% to 62.8% of alleles exhibited ASE in the same condition and 34.7% of alleles showed ASE among different tissues or conditions [32]. In hybrid *Camellia sinensis*, only 10.4% of alleles exhibited consistent ASE genes among tissues [44]. Therefore, investigating NAE and ASE patterns in hybrids is crucial for unraveling the molecular mechanisms underlying heterosis. These patterns provide insight into how genetic and molecular interactions contribute to the enhanced performance of hybrid progeny.

### 2.3. Epigenetic Regulation of Heterosis

**Figure 2:**
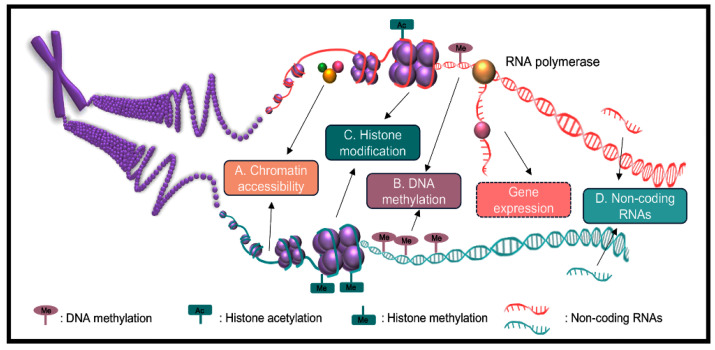
Epigenetic regulation of heterosis. (**A**). Chromatin accessibility. (**B**). DNA methylation. (**C**). Histone modification. (**D**). Non-coding RNAs.

Hybridization often induces global changes in epigenetic modifications, which in turn alter gene expression. Increasing evidence suggests that epigenetic regulation plays a pivotal role in the formation of heterosis [32,43,44,45,46]. Epigenetic modifications (Figure 2), including chromatin accessibility, DNA methylation, histone modifications, and non-coding RNAs, are essential for plant development, evolution, and environmental adaptation [47,48]. These modifications influence gene regulatory networks and contribute to the enhanced performance observed in hybrid progeny.

Chromatin accessibility refers to the physical availability of regulatory proteins to cis-regulatory elements and is positively associated with gene expression levels (Figure 2A) [49]. In a study of inter-subspecific hybrid *O. sativa* and its parental lines, it was observed that the paternal genome contributes more chromatin accessibility regions (ACRs) to the hybrid offspring [46]. Furthermore, the number of upregulated ACRs in the hybrid surpasses the number of downregulated ones, suggesting an enhanced regulatory capacity in the hybrid, offering a novel perspective for understanding heterosis [46]. Similarly, in *Camellia sinensis* hybrids, the increased number of ACRs compared to their parental lines regulates ASE patterns and influences metabolite formation [50].

DNA methylation, specifically the methylation of cytosine at the fifth position, is a key regulator of gene expression and plays a critical role in plant development and adaptation to environmental changes (Figure 2B) [51]. In hybrid *Glycine max*, DNA methylation reprogramming has been linked to phenotypic variation and heterosis, highlighting its importance in hybrid vigor [52]. Similarly, studies in *Arabidopsis* F1 hybrids have demonstrated that DNA methylation contributes to the regulation of heterosis [53,54]. Further research has revealed that heterosis is more strongly influenced by *DDM1*-mediated DNA methylation than by *MET1*-mediated maintenance of methylation [55,56]. Additionally, in *Hibiscus cannabinus* F1 hybrids, lower levels of DNA methylation regulate gene expression, enhancing cadmium tolerance heterosis [57].

Histone modifications, such as methylation, acetylation, and phosphorylation, regulate plant development and stress resistance (Figure 2C) [58]. Comparative studies of histone modifications in hybrid *O. sativa* and their parents found that H3K36me3, rather than H3K27me3, primarily regulates ASE in hybrids [59]. A study on Ler/C24 hybrids and their parents showed that H3K4me3, H3K9ac, H3K27me3, and H3K9me2 participate in the regulation of specific gene expression in hybrids [60]. For example, H3K4me3 is positively correlated with differentially expressed genes and promotes biomass-related heterosis in hybrids [61]. In *Arabidopsis* F1 hybrids, histone modifications finely tune *CCA1* expression, enhancing pathogen defense while maintaining growth, thereby promoting biomass-related heterosis under pathogen infection [62].

Non-coding RNAs (ncRNAs) are RNA molecules that do not code for proteins but play critical roles in regulating gene expression, chromatin structure, and translation (Figure 2D) [63]. Analyzing inter-subspecific hybrid *O. sativa* and their parental lines revealed that ncRNAs exhibiting non-additive expression, along with ASE-related ncRNAs, are pivotal in *O. sativa* heterosis [45]. Research indicates that small interfering RNAs (siRNAs) contribute to chromatin maintenance and genome stability, while variations in microRNA (miRNA) expression can influence growth vigor and adaptability [64]. In *Piper nigrum* hybrids, specific miRNAs, such as miR156 and miR169, have been identified as important regulators of heterosis, underscoring the essential role of ncRNAs in hybrid vigor across different plant species [65].

## 3. Prediction of Heterosis

Conducting numerous hybridization experiments to obtain progenies exhibiting heterosis requires substantial resources and time, and often yields unreliable results [66]. The key to efficiently utilizing heterosis is the accurate prediction and selection of optimal hybrid combinations. Research indicates that combining ability can serve as a basis for predicting heterosis [67]. Combining ability is an important metric in plant breeding that is used to select superior parental lines and predict heterosis. Chen et al. (2019) [68] performed a genome-wide association study (GWAS) on general combining ability (GCA) in *O. sativa*, finding that advantageous alleles (*Ghd8*, *GS3*, and *qSSR4*) accumulated in high-GCA parents, explaining approximately 30% of GCA yield variations. Studies have shown that less than 10% of parental lines contain all of these three advantageous alleles, and molecular-assisted selection can quickly eliminate parents lacking these genes, saving significant time and labor [68]. A GWAS of specific combining ability (SCA) identified 12 loci associated with dominant agronomic traits, confirming the role of combining ability in heterosis prediction [68].

Genetic distance refers to the degree of genetic difference between different populations or species [69]. Higher genetic diversity is generally thought to contribute to greater heterosis in offspring, so when selecting parents, taking into account the genetic distance between them can optimize heterosis traits in offspring. Assessing genetic distance using molecular markers facilitates the rapid prediction of heterosis [69,70,71]. Research has demonstrated that employing SNPs in heterozygous promoter regions can effectively identify inbred lines with high heterotic potential, thereby enhancing breeding efficiency [72]. Additionally, Geng et al. (2021) [73] found that SSR and SNP markers can be utilized to evaluate heterosis in *Gossypium hirsutum* parents, aiding in the prediction of key traits such as fiber length and fiber elongation rate.

Likewise, omics approaches, including genomics, transcriptomics, and metabolomics, can also be employed to predict heterosis [74,75,76]. Huang et al. (2015) [74] constructed a genomic map for 1495 elite hybrid *O. sativa* lines and their near-isogenic lines, revealing that the accumulation of numerous rare advantageous alleles contributes to heterosis, laying a foundation for heterosis prediction models for *O. sativa* yield. Fu et al. (2023) [75] identified candidate genes such as *OsDCL2*, *Pi5*, *DTH8*, and *Hd1* through transcriptomic and metabolomic analyses that can be used to predict super-parental heterosis. Xu et al. (2016) [76] analyzed the metabolomes of 21,945 hybrid *O. sativa* lines and found that the top ten predicted hybrids could increase yield by approximately 30%, highlighting the powerful role of metabolomics in heterosis prediction.

In summary, population stratification by MAS and various omics approaches was used to assess parental combining ability and genetic distance, which can effectively select optimal hybrid combinations, facilitating the development of superior hybrids.

## 4. Advances in Heterosis Research in Tree Breeding

Heterosis has been extensively studied in woody species, leading to significant advancements in growth traits, resistance to disease and abiotic stresses, and timber volume of hybrid progeny in forestry [8]. For instance, hybrid poplar “84K” (*Populus alba* × *P. tremula* var. *glandulosa*) exhibits accelerated growth and enhanced adaptability to various environmental conditions compared to its parent species [77]. Triploid hybrid *Salix* demonstrates superior hybrid vigor in terms of harvestable biomass and associated growth traits [78]. Retief et al. (2009) [79] demonstrated that dominant-expressed genes play a crucial role in the wood volume growth vigor of hybrid eucalyptus progeny, while dominant genes in the hybrid progeny of *Eucalyptus grandis* and *E. urophylla* can overshadow recessive gene effects, thus fostering hybrid vigor [80]. Ren et al. (2024) [37] found that a large number of genes associated with hormone signal transduction and related transcription factors such as MYB88, LHU, and TCP4 may promote the formation of heterosis.

Plants exhibiting heterosis often possess high levels of genome heterozygosity, highlighting the significance of high-resolution genome assembly in elucidating the molecular mechanisms underlying heterosis. Recent advancements include the sequencing of haplotype genomes for various tree species, such as hybrid *Populus* [32,81,82,83,84,85], *Pyrus* [86], *Malus demestica* [87], *Morus atropurpurea* [88,89], *Carya illinoinensis* [90], and *Actinidia zhejiangensis* [91]. Utilizing these genomic resources could excavate allelic sequence variation, and alongside multi-omics approaches, could resolve the differences between epigenetic modification and gene expression of alleles. This has yielded significant insights into the molecular mechanisms of heterosis in forestry. For instance, Shi et al. (2024) [32] identified extensive expression differences among alleles in the 84K poplar, revealing that gene body CHG methylation, sequence divergence, and transposon occupancy near alleles are critical factors influencing ASE. Luo et al. (2024) [85] attributed the high hybrid vigor of Nanlin 895 poplar to its markedly higher genome heterozygosity and increased genomic variation compared to other poplar lines. ASE analysis indicated that many alleles in Nanlin 895 exhibit variability across different tissues or environments [85]. The *PtoNF*-*YC9*-*SRMT*-*PtoRD26* regulatory module contributes to the enhanced salt tolerance and hybrid vigor observed in triploid white poplar [82]. The non-additive expression of circular RNAs and parent-of-origin circular RNAs has been shown to regulate hybrid vigor in poplars [92]. In pears, the contribution of allele-specific expression to hybrid vigor has been highlighted, particularly regarding fruit quality traits, with ASE involvement in wax accumulation and consistent ASE expression enhancing sugar content [86]. In apples, ASE genes were identified in the “M9” and “MM106” varieties associated with stress tolerance, noting that *DwTE* insertion is closely related to the dwarfing trait in “M9” [87]. Transcriptomic analysis of Chinese fir (*Cryptomeria japonica*) revealed that high productivity in super-parental hybrids is associated with 14 upregulated genes significantly stimulated by environmental factors, thus enhancing hybrid growth vigor [93]. Additionally, transcriptomic analysis of rubber trees indicated that over-dominant expression genes in rubber seedlings significantly contribute to growth vigor [94]. Further analysis of hybrid rubber and its parent transcriptomes showed that high yield in F1 hybrids correlates with genome heterozygosity, with whole-genome genetic variation being crucial for maintaining hybrid vigor [95]. Integrated transcriptomic and metabolomic analyses of Zhongshanhanxiao (*Michelia formosana*) demonstrated that candidate genes related to cell division, pathogen resistance, and organic accumulation regulate its growth vigor [96]. Despite substantial progress in understanding hybrid vigor according to genetic structure variation, epigenetic differences, and gene expression perspectives, there remains a need for comprehensive analyses addressing wood growth rate, cell wall deposition, tree-specific traits, and tree adaptability to environmental conditions.

## 5. Application of Tree Genomics in Unraveling Heterosis Mechanisms

Tree genomics plays a pivotal role in enhancing productivity, adaptability, resilience, and sustainability [97]. Over the past two decades, advances in single-cell transcriptomics, single-cell ATAC-seq, spatial transcriptomics, machine learning, CRISPR-mediated genome editing, and bioinformatics have accelerated the development of tree genomics. These technologies have generated a wealth of multi-dimensional spatiotemporal gene expression data, significantly contributing to the elucidation of the molecular mechanisms underlying phenotypic variation and genetic diversity.

Single-cell RNA sequencing (scRNA-seq) has become a powerful tool for studying organ formation and tissue development. For instance, scRNA-seq has been applied in trees such as *Populus* [98,99,100,101], *Eucalyptus grandis* [98], *Liriodendron chinense* [98], and *Trochodendron aralioides* [98], which revealed gene regulatory networks involved in cambial differentiation, xylem development, and phloem development during wood formation [98,99,100,101]. Furthermore, scRNA-seq has been applied to detect the asymmetric expression of homologous genes during various plant developmental processes [102,103,104]. Thus, leveraging scRNA-seq to analyze cell-type-specific asymmetric expression pattern alleles during hybrid progeny development can resolve key genes and signaling pathways associated with heterosis. Moreover, single-cell ATAC sequencing (scATAC-seq) is pivotal for elucidating cell-type-specific regulatory networks during plant development [104,105]. This technique can be employed to dissect cell-type-specific asymmetric allelic epigenetic modifications and regulatory networks that govern heterosis formation at the single-cell level.

Recently, spatial transcriptomics has been successfully used to map gene expression profiles and transcriptional networks involved in cambial differentiation into secondary vascular tissues during stem development in poplars [106,107]. Combining high-resolution haplotype genomes with spatial transcriptomics allows for the study of allele-specific expression patterns at the spatial level, identification of key alleles, and interactions between different cell types and how these interactions affect the formation of heterosis.

Machine learning models are increasingly applied to investigate plant molecular mechanisms. For example, ASE patterns have been predicted with high accuracy using machine learning, identifying DNA methylation, transposon occupancy, and sequence differentiation as key regulatory factors [32]. Predictions of 70,000 different gene editing strategies revealed that the top seven strategies reduced lignin content by 35%, leading to the development of superior tree varieties with up to 32% reduced lignin through CRISPR editing [108]. These studies suggest that integrating multi-omics analyses with CRISPR tools can facilitate a comprehensive understanding of hybrid vigor mechanisms by investigating key alleles at both the molecular and spatial levels.

## 6. Conclusions and Future Perspectives

The utilization of heterosis has revolutionized forestry; however, theoretical research in this area remains relatively underdeveloped, limiting further applications in forest production practice. Currently, dominance, overdominance, and epistasis effects are considered the main hypotheses explaining heterosis. Nevertheless, these hypotheses have limitations and do not fully explain the genetic mechanisms underlying heterosis. The formation of heterosis is a dynamic process influenced by developmental stages and environmental conditions, but existing research often focuses on specific developmental stages and may not fully represent overall performance.

Future research should employ multi-omics approaches to conduct comprehensive, multi-dimensional studies of F1 hybrid progeny and their parents, aiming to construct gene regulatory networks for heterosis formation. Additionally, integrating CRISPR tools to study allele functions and identify hybrid vigor loci is essential. In conventional breeding, breeders often test numerous hybrid combinations to identify optimal ones, yet only a small fraction produce high-quality hybrids. Therefore, the selection of the parent with the most heterosis-related loci for hybridization is crucial for efficiently harnessing heterosis and avoiding the blind testing of numerous combinations. Once heterosis loci are confirmed, molecular marker-assisted technologies can be used to analyze key loci genotypes across parent lines, narrowing down potential high-performing combinations. This approach will significantly reduce the labor and time costs associated with hybrid breeding in tree species.

## Data Availability

Not applicable.
